# Innovation in a Continuous System of Distillation by Steam to Obtain Essential Oil from Persian Lime Juice (*Citrus latifolia* Tanaka)

**DOI:** 10.3390/molecules26144172

**Published:** 2021-07-09

**Authors:** José Daniel Padilla-de la Rosa, Magaly Dyanira Manzano-Alfaro, Jaime Rosalío Gómez-Huerta, Enrique Arriola-Guevara, Guadalupe Guatemala-Morales, Anaberta Cardador-Martínez, Mirna Estarrón-Espinosa

**Affiliations:** 1 Unidad Zapopan, Centro de Investigación y Asistencia en Tecnología y Diseño del Estado de Jalisco (CIATEJ), Av. Camino Arenero No. 1227, Col. El Bajío, Guadalajara C.P. 45019, Mexico; jdpadilla@ciatej.mx; 2 Departamento de Ingeniería Química, CUCEI, Universidad de Guadalajara, Blvd. Marcelino García Barragán #1421, esq. Calzada Olímpica, Guadalajara C.P. 44430, Mexico; magalister_26@hotmail.com (M.D.M.-A.); arriole@hotmail.com (E.A.-G.); 3 Departamento de Química, CUCEI, Universidad de Guadalajara, Blvd. Marcelino García Barragán #1421, esq. Calzada Olímpica, Guadalajara C.P. 44430, Mexico; jarogohue@hotmail.com; 4 Unidad Normalistas, Centro de Investigación y Asistencia en Tecnología y Diseño del Estado de Jalisco (CIATEJ), Av. Normalistas No. 800, Guadalajara C.P. 44720, Mexico; 5 Escuela de Ingeniería y Ciencias, Tecnologico de Monterrey, Epigmenio González 500 Fracc. San Pablo, Querétaro C.P. 76130, Mexico

**Keywords:** essential oils, distillation by steam, continuous processes

## Abstract

The citrus industry is one of the most important economic areas within the global agricultural sector. Persian lime is commonly used to produce lime juice and essential oil, which are usually obtained by batch distillation. The aim of this work was to validate a patented continuous steam distillation process and to both physically and chemically characterize the volatile fractions of essential Persian lime oil. Prior to distillation, lime juice was obtained by pressing the lime fruit. Afterwards, the juice was subjected to a continuous steam distillation process by varying the ratio of distillate flow to feed flow (0.2, 0.4, and 0.6). The distillate oil fractions were characterized by measuring their density, optical rotation, and refractive index. Gas chromatography GC-FID was used to analyze the chemical compositions of the oil fractions. The process of continuous steam distillation presented high oil recovery efficiencies (up to 90%) and lower steam consumption compared to traditional batch process distillation since steam consumption ranged from 32 to 60% for different steam levels. Moreover, a reduction in process time was observed (from 8 to 4 h). The oil fractions obtained via continuous steam distillation differed significantly in their composition from the parent compounds and the fractions.

## 1. Introduction

*Citrus* is the most abundant crop of fruit trees around the world, with annual production in 2015 totaling approximately 130,947 million tons. The main citrus fruits grown globally are oranges, mandarins, lemons, limes, and grapefruits. Brazil, China, India, Mexico, Spain, and the USA produce over two-thirds of the world’s citrus fruits [1]. In 2017, the worldwide production of acid lime fruit was 17,218,173 tons, among which Mexico contributed 2,528,174 tons and was thus considered the main producer of lime fruit, comprising 14.7% of the total production [2].

### 1.1. Industrial Use of Lemons

For many years, the citrus processing industry has focused on the production of juices and essential oils. It was estimated that 33% of citrus harvested in the world is used to produce juices [3]. In the processing of citrus fruits (orange, lemon, lime, grapefruit, etc.) for juice and essential oil extractions, peels represent between 50 and 65% of the total weight of the fruits and remain the primary byproduct [4]. This solid residue, referred to as citrus waste, is estimated to total 15 million tons per year worldwide [5]. Although citrus peels are used as cattle feed and as a source of essential oils, the correct management of this residue remains an ongoing problem for the citrus industry [3]. The major components of dry citrus peel waste include sugars (23%), cellulose (22%), pectin (25%), hemicellulose (11%), flavonoids (4.5%), and up to 4% essential oil, often referred to as citrus essential oil [6].

### 1.2. Production of Essential Oils

Different industries appreciate citrus essential oils because of their pleasant aroma and incredible variety of applications [7]. In the food industry, citrus essential oil is used as the main aromatic ingredient in non-alcoholic carbonated drinks, cola drinks, pastries, sweets, and biscuits. In the pharmaceutical industry, this type of essential oil is used as a flavoring to mask the unpleasant taste of drugs [8]. In the chemical industry, these oils are used as fragrances in air fresheners and cleaning products. In the perfume industry, citrus essential oils are used as the primary raw materials to formulate toiletries and perfumes [7].

Citrus oil generally contains over 90% terpenes, about 5% oxygenated compounds, and less than 1% non-volatile compounds, such as waxes and pigments [9,10]. Essential oils consist of mixtures of volatile and non-volatile components, such as terpenes, sesquiterpenes, higher alcohols, aldehydes, ketones, acids, esters, camphor, and waxes.

The volatile fraction of Persian lime oil is characterized by higher levels of d-limonene, γ-terpinene, esters, and monoterpene aldehydes and lower amounts of β-pinene + sabinene sesquiterpenes and aliphatic aldehydes than key lime oils [11]. Interestingly, although d-limonene comprises more than 90% *w/w* of the volatile composition of the essential oil, its contribution to the final aroma of the mixture is limited by its instability and the possibility of forming off-flavor compounds [7].

**Figure 1:**
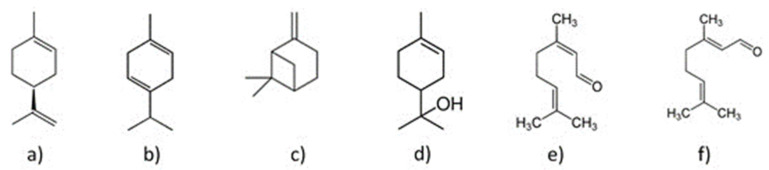
Compounds in the essential oil of Persian lime: (**a**) d-limonene, (**b**) γ-terpinene, (**c**) β -pinene, (**d**) α-terpineol, (**e**) neral, and (**f**) geranial.

Some of the representative compounds in the essential oil from Persian lime are shown in Figure 1.

### 1.3. Types of Essential Oils

There are different types of essential oils depending on the process used to obtain them, including cold-pressed oil, distilled oil, concentrated oil, centrifuged essential oil, and juice oils [12].

Centrifuged oil is classified based on the extraction technology used [11]. Oil type A is obtained via centrifugation of the oil/juice emulsion produced by screwing the whole fruit in a press that crushes the fruit. The traditional method to extract essential oils is by cold pressing the citrus peels. The oil is present in oil sacs or oil glands located at different depths in the peel and cuticles of the fruit. Peel and cuticle oils are removed mechanically by cold pressing. Since cold pressing yields a watery emulsion, this emulsion is then centrifuged to separate out the essential oil [13]. Oil type B is obtained by rasping the peel to release the oil [11].

Because of its high solubility and pronounced flavor characteristics, distilled essential oil is preferred for the manufacture of beverages [14], both alcoholic and non-alcoholic, especially cola beverages.

The production of essential oil on a commercial scale is carried out using traditional methods such as steam distillation [6]. This kind of distillation is performed in batches. High temperatures are used for distillation, and the energetic consumption makes this process expensive. Moreover, high temperatures could induce degradation during distillation. Lime oil distilled from crushed lime fruit is rich in terpene alcohols and low in aldehydes and bicyclic hydrocarbons compared to pressed lime oil. During the production of distilled lime oil, acid-catalyzed reactions of bicyclic hydrocarbons occur, increasing the content of terpene alcohols, especially α-terpineol. Terpene aldehydes are almost completely lost during distillation [9]. These conventional methods show some disadvantages related to high energy costs and long extraction times [6].

During distillation, the citrus peels are exposed to boiling water or steam, releasing their essential oils through evaporation. As steam and essential oil vapors are condensed, both are collected and separated in a vessel traditionally called the “Florentine flask” [13]. Distillation from lemon peel provides an oil yield of 0.21% from the total peel weight compared to 0.05% when using cold pressing [1].

### 1.4. Innovative Technologies

To reduce the use of extractive solvents and the required energy, novel techniques are now available, including the use of supercritical extraction steam explosion, accelerated extraction at a high temperature and pressure, micro-waves, and ultrasound-assisted extraction [15].

The “Green Chemistry” concept is encouraging the development of these new environmentally friendly techniques [15]. Although the above technologies have several advantages, they also require more sophisticated levels of technology that entail higher investment costs. These costs may not be easily attainable at industrial levels for average small- and medium-sized companies that extract essential oils in underdeveloped or moderately developed countries.

Hence, the industry requires the development of alternative technologies with reduced energy consumption based, e.g., on the basic principle of steam entrainment distillation, which can be incorporated quickly and easily in industrial processes either through incorporation into or the adaptation of existing technologies. This methodology has implications for business success since the possible replacement of existing technology entails a sometimes-radical paradigm shift involving large investments of time and money. In this sense, continuous steam entrainment distillation could be a technological option for traditional steam entrainment processes.

### 1.5. Continuous Distillation by Steam

**Figure 2:**
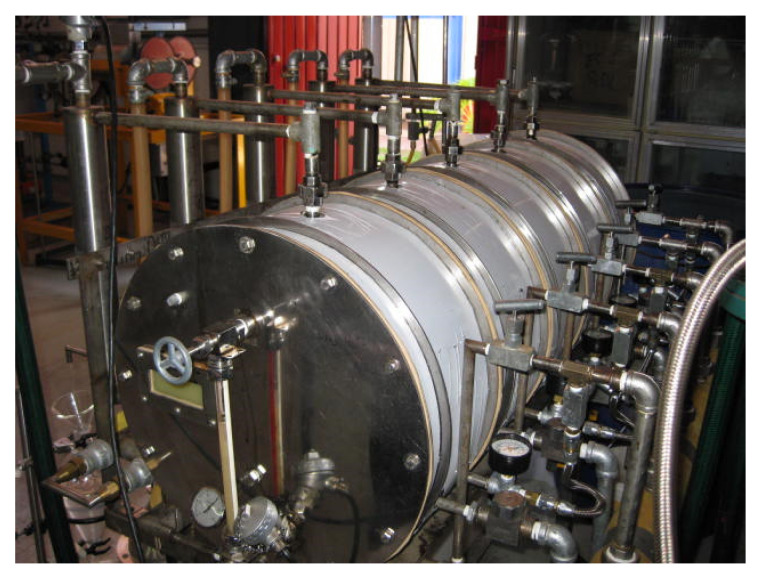
Multifunctional distillation prototype.

The fractionation of essential oils rich in functional groups is a challenge with great prospects for the food and pharmaceutical industries because of the diverse range of medicinal properties belonging to these oils. To this end, the Centro de Investigación y Asistencia en Tecnología y Diseño del Estado de Jalisco developed a patented continuous steam distillation process that can be used to obtain essential oil in a more economical form than the traditional process of batch distillation [16] (Figure 2).

Continuous distillation is an economic process that presents opportunities and challenges for the fractionation of essential oils.

Research on continuous distillation was previously focused on the steam distillation of essential oils from a Mexican lime lab prototype at the level of 3 L [17] and recently from the rectification of a tequila pilot facility at the level of 60 L [18].

The continuous steam distillation process has many advantages and benefits over the batch distillation process. For example, vapor consumption in continuous steam distillation saves up to 50% energy over batch distillation, and a potential fractionation of essential oil can be obtained. The residual juice can then be used to formulate a hydrating drink. These advantages result in a better oil quality because, in traditional distillation, the juice’s long contact times at high temperatures and acidic pH levels cause the degradation of oxygenated compounds [14].

### 1.6. Continuous Distillation Process of Essential Oils

The operational procedure for the continuous distillation equipment is as follows: The juice flow is fed (F), passing successively through each of the five stages, thus obtaining a residue flow (W). In each of the stages, a steam stream (S) is fed through a diffuser, which rises in the form of bubbles, in which the distilled essential oil emulsified in the lemon juice is diffused. Then, the obtained steam (V) is condensed in each of the condensers of each stage, thereby obtaining the distillate stream (D), where the distilled oil is separated via decantation for subsequent physicochemical and chromatographic analysis.

**Figure 3:**
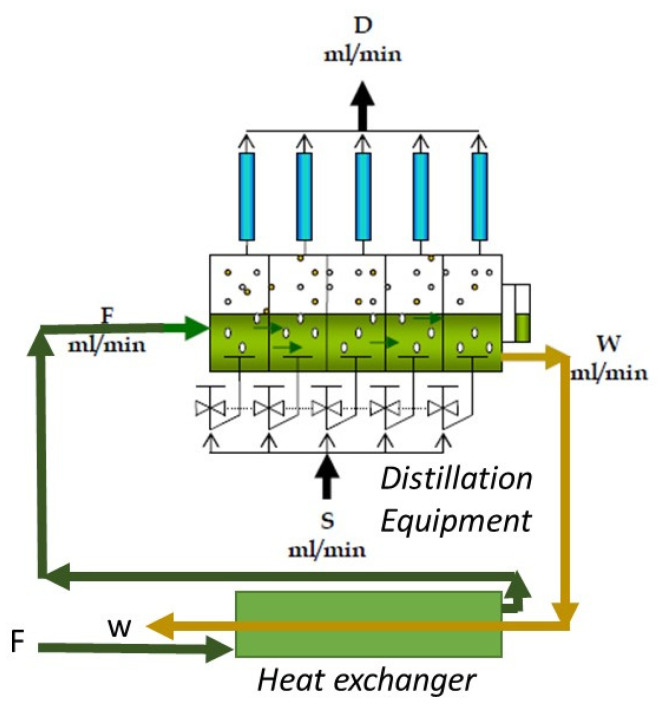
Schematic figure of distillation.

The liquid to be distilled is fed (F) in the first stage (Figure 3) and travels through each of the five stages of the equipment until obtaining the waste stream (W). In each of the steps, both heat exchangers carry out evaporation of the volatiles, which are recovered in each of the five condensers to obtain the distillate (D).

This equipment has a heat recovery system that allows the recovery of the heat of the waste (W) from preheating the feed (F), which achieves additional energy savings when recovering the sensible heat. In each of the condensers, a distilled fraction (D) is obtained, which is then characterized. According to the desired profile or composition, this fraction (D) can be mixed [18].

The aim of this work was to validate a continuous steam distillation process (in terms of recuperation and energy efficiency) and to characterize the volatile fractions of essential Persian lime oil both physically and chemically.

## 2. Results and Discussion

### 2.1. Content of Essential Oil in Citrus Juice

The fruit of Persian citrus (*Citrus latifolia* Tanaka) contained 52% juice and 0.984 ± 0.287% *v/v* essential oil content. The essential oil recovered in the batch process was 94.7% ± 2.34, whereas that recovered through continuous distillation was 96.14% ± 2.32.

### 2.2. Efficiency of the Process and Steam Consumption in Continuous Distillation

**Figure 4:**
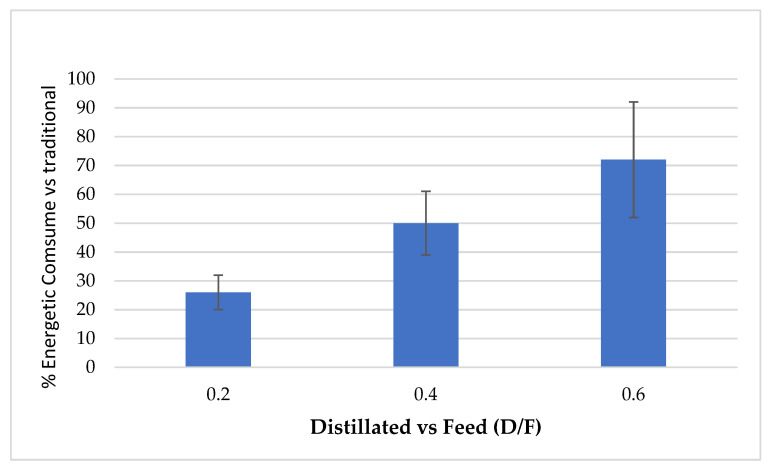
Percentage of steam consumption in continuous vs. traditional processes.

The continuous distillation process presented high oil recovery efficiency (up to 90%) and lower steam consumption (Figure 4) compared to traditional batch process distillation since steam consumption ranged from 32 to 60% for different steam levels (D/F).

### 2.3. Physicochemical Analysis

The variance analysis showed only the fraction number to have a significant effect on the refractive index, density, and optical rotation, whereas the distillate vs. feed (D/F) showed no significant effect (Table 1).

**Figure 5:**
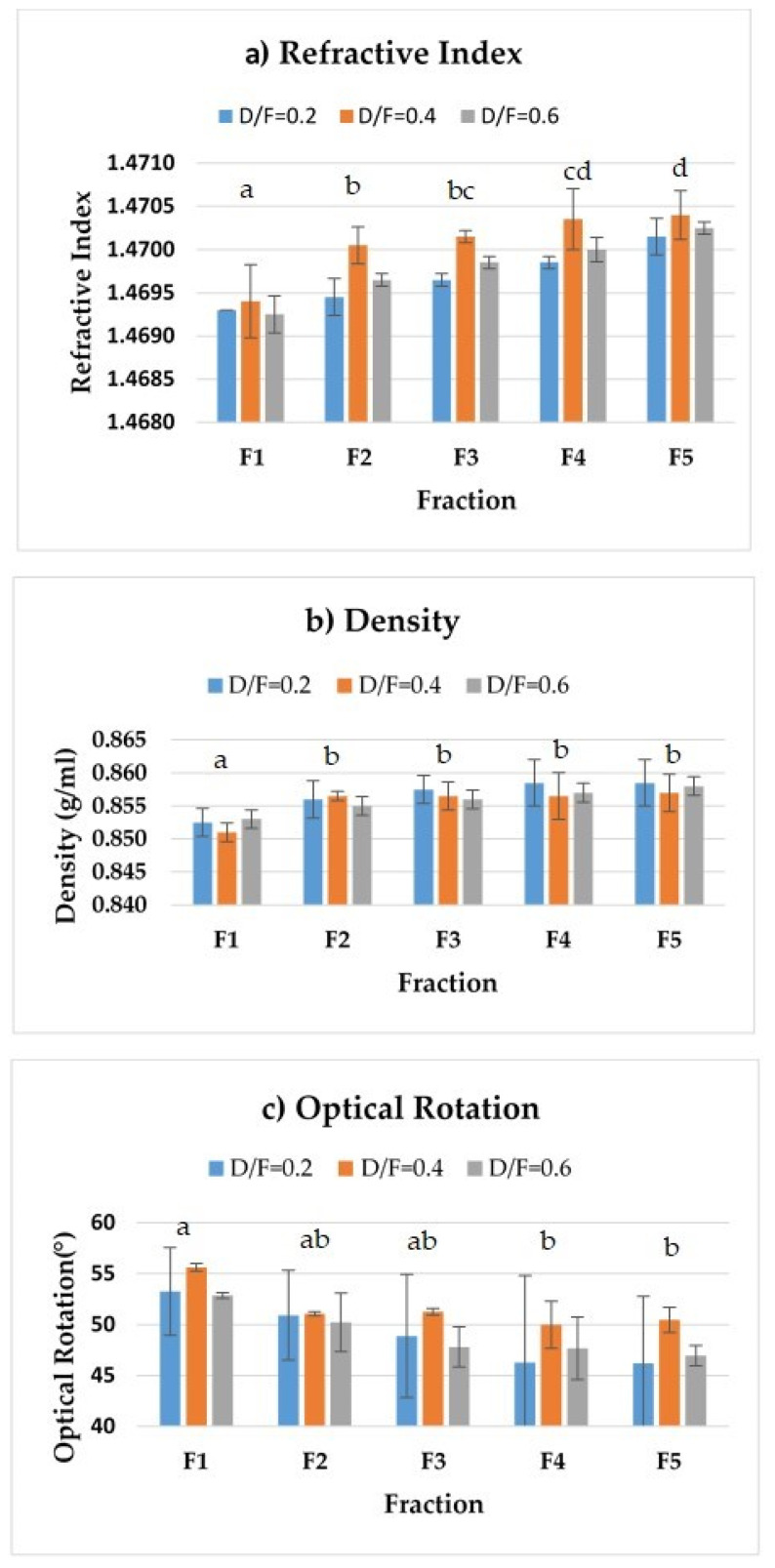
Physical properties: (**a**) refractive index, (**b**) density, and (**c**) optical rotation. The results are the average of three replicates ± standard deviation. Different letters over a column indicate statistical differences (Tukey, *p* < 0.05).

The density and refractive index profiles showed an increase, whereas the optical rotation profile showed a decrease, in the fractions distilled at the three dosage levels of steam (Figure 5).

The increase in the refractive index density (from 1.4693 to 1.47015) of the distilled fractions (from 0.8525 to 0.8585 g/mL) indicated a change in the composition of those fractions. Whereas F1 contained lighter compounds (terpenes), F5 contained heavier compounds (oxygenated). On the other hand, the observed decrease in optical rotation was linked to a reduction in the content of d-limonene, resulting in average values of +53.25° up to +46.2° for a D/F of 0.2 (Table 2).

#### 2.3.1. Effect of the Fraction Number on the Density and Refractive Index of Essential Oil

We observed significant differences (*p* < 0.05) in the refractive index and density of essential oil depending on the fraction number. Fractions were grouped by refractive index into four groups. The first group corresponded to Fraction 1, the second group included Fractions 2 and 3, and the third group included Fractions 3 and 4. Finally, fraction four included Fractions 4 and 5.

The refractive index of the reference reported in the literature is 1.4751–1.4743 [19]. These values are higher than those obtained by the fractions (Figure 5a), which may be because the residence time we explored was four hours, whereas the traditional process uses eight hours of contact time between the juice and the steam under acidic conditions, thereby impacting differences in the composition.

For density (Figure 5b), there were two homogeneous groups. The first (group a) included Fraction 1 and the second (group b) included Fractions 2, 3, 4, and 5. The density of the reference reported in the literature [19] is 0.8579–0.8556 g/mL whose range of values was close to that of Fraction 3 (Table 2).

For optical rotation, there were two homogeneous groups (Figure 5c. The first (group a) included Fractions 1, 2, and 3, and the second (group b) included Fractions 2, 3, 4, and 5. The optical rotation of the reference reported in the literature (Table 2) is +50.52°–+46.84°, whose range of values was close to that of Fraction 3.

Usually, commercially available distilled essential oil presents similar physical–chemical properties (in terms of density and optical rotation) to those of F3 (Table 2).

With 95% confidence, no statistically significant differences were found in the physicochemical parameters (refractive index, optical rotation, and density) between fractions 1, 2, 3, 4, and 5 and the oil obtained by batch distillation. Compared to the reference, the only parameter that showed significant differences was the refractive index (Table 2). According to Dugo et al. [11], the refractive index of Persian lime essential oil obtained by cold pressing was 1.4818, whereas the optical rotation was 47.3 degrees. The Persian lime essential oil obtained by hydro-distillation had similar values for the refractive index, the optical rotation, and the density than cold pressing or the fractions obtained by continuous steam distillation [14,19]. These results suggest that thermal treatment does not change the physicochemical properties of essential oils but possibly the composition. However, it is necessary to conduct further studies varying the residence time (4 h) of oil inside the continuous steam distillatory system over its physicochemical characteristics.

#### 2.3.2. Effect of D/F on Density, Optical Rotation, and Density

**Figure 6:**
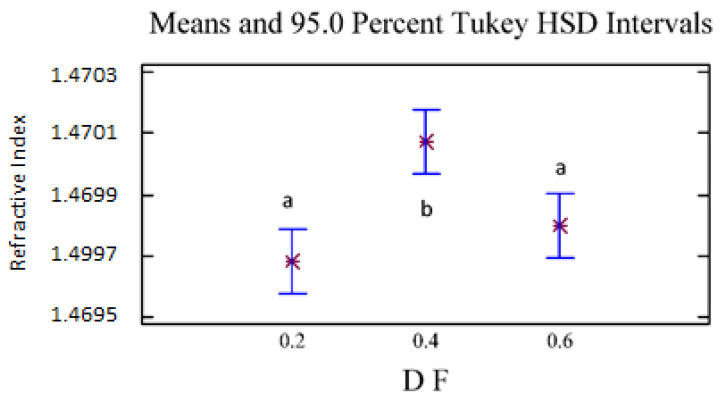
Refractive Index vs. D/F. The results are the average of three replicates ± standard deviation. Different letters over a column indicate statistical differences (Tukey, *p* < 0.05).

The refractive index and the optical rotation presented statistically significant differences with 95% confidence because of the D/F effect. For density and optical rotation, no statistically significant differences were found because of the D/F relationship. For the refractive index (Figure 6), we observed two homogeneous groups: the first homogeneous group (group a) included D/F ratios of 0.2 and 0.6, and the second group (group b) included a D/F ratio of 0.4.

### 2.4. Gas Chromatography for Key Compounds

Analysis by gas chromatography permitted to identify an average of 47 compounds, either in the batch-distilled essential oil or in the fractions obtained by continuous steam distillation. Usually, the number of compounds that can be identified in citrus essential oil varies among 30 compounds in citrus essential oil obtained by hydro-distillation [13]. Simas et al. [20] reported a total of 38 compounds in citrus essential oil also obtained by hydro-distillation.

On the other hand, Dugo et al. [11] reported 66 compounds in industrial cold-pressed essential oil of Persian lime. The higher number of compounds identified could be related to thermal treatment. Moreover, these authors obtained a higher number of compounds in fractionated Persian lime essential oil. However, the fractions were obtained by glass column chromatography [11]. Although more compounds can be obtained by this method, it is more time-consuming compared to continuous steam distillation.

No matter the method used to obtain essential oils from citrus species, the most abundant compound is d-limonene. Other abundant compounds are β-pinene, geranial, neral, p-cymene, and α-terpineol, which contribute significantly to the final composition of essential oil [9,13,20]. Taking advantage of the abundance of these compounds, they were used to analyze the effect of continuous steam distillation on the composition of essential oil fractions.

The D/F experimental ratio and fraction number had a very significant effect on the chemical profiles of the distilled fractions with 95% confidence, as shown in Table 3 for d-limonene, p-cymene, α-terpineol, β-pinene, neral, and geranial. For γ-terpinene, only the factor of the fraction number was significant at 95%, whereas the effect of the D/F relationship was not significant. Consequently, the chemical composition was significantly different in the distilled fractions (Table 3).

#### 2.4.1. Effect of the Fraction Number on Relative Abundance

**Figure 7:**
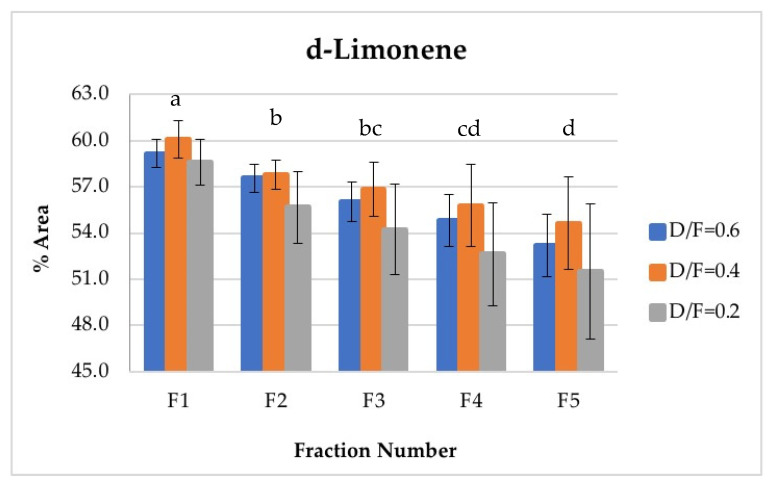
Abundance profile vs. number fraction for d-limonene. The results are the average of three replicates ± standard deviation. Different letters over a column indicate statistical differences (Tukey, *p* < 0.05).

The d-limonene, citral (neral + geranial), and β-pinene showed a decrease in composition (% of area) depending on the number fraction (Figures 7, 10, 12 and 13). Conversely, the p-cymene and α-terpineol concentrations showed an increase as the distillation time progressed (Figures 9 and 11).

D-limonene is the most abundant and volatile component in lime essential oil. Therefore, d-limonene was the most abundant component in the vapor phases of all fractions obtained and was present in the greatest abundance during the first phase. The composition of d-limonene tended to reduce to approximately the fraction number, from an average content of 59.28% in the first fraction to an average content of 53.12% in the fifth fraction (Figure 7). In this way, Fractions 1 and 2 featured higher d-limonene content than that obtained by the batch process, whose content was similar to that of Fraction 3. Conversely, Fractions 4 and 5 presented lower d-limonene content, with average values of 54.41% and 53.12%, respectively, in percentage of area. In the multiple range test, three homogeneous groups were obtained: a (containing F1), b (containing F2 and F3), and c (containing F3, F4, and F5).

**Figure 8:**
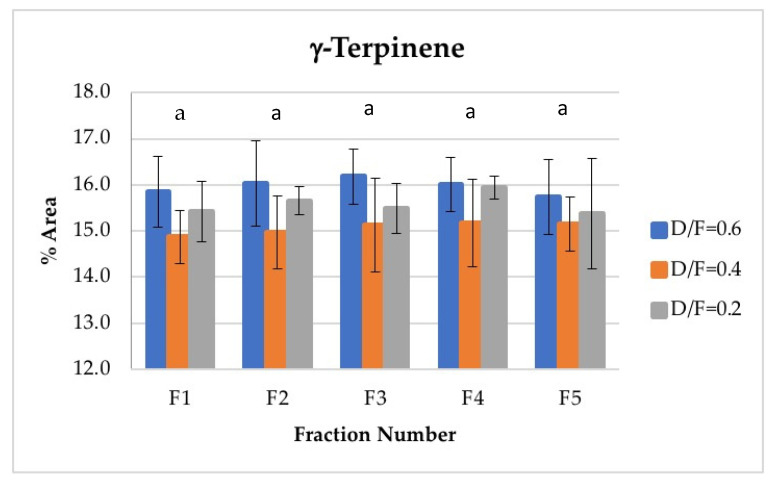
Abundance profile vs. number fraction for γ-terpinene. The results are the average of three replicates ± standard deviation. Different letters over a column indicate statistical differences (Tukey, *p* < 0.05).

For γ-terpinene, the second most abundant terpene, no significant differences were found in its abundance because of the effect of the fraction number. This is an interesting result given that γ-terpinene is chemically transformed to p-cymene during steam distillation and that p-cymene increases its abundance as a function of the fraction number. γ-terpinene may not have decreased in concentration because of its γ-terpinene range. On the one hand, γ-terpinene concentrations were much higher than those of p-cymene. However, this abundance was mainly attributable to the fact that γ-terpinene was produced during distillation from d-limonene, which is a d-limonene isomer and thus the most abundant component observed in the oil (Figure 8).

**Figure 9:**
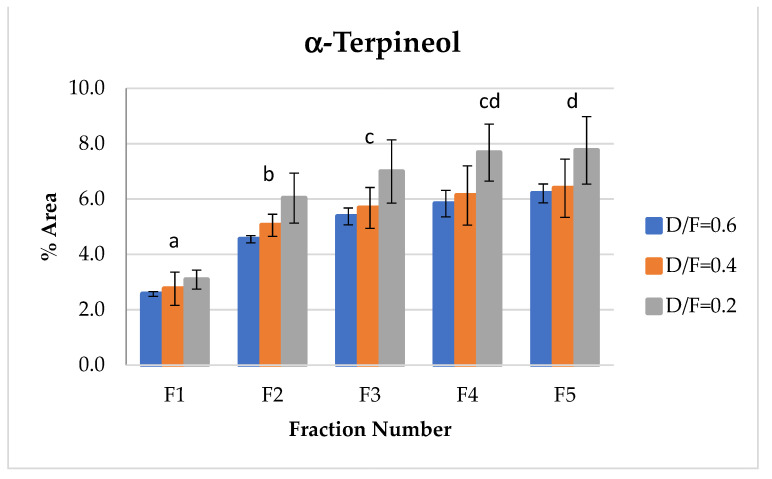
Abundance profile vs. number fraction for α-terpineol. The results are the average of three replicates ± standard deviation. Different letters over a column indicate statistical differences (Tukey, *p* < 0.05).

α-terpineol, one of the representative alcohols in essential oil, showed an increase in abundance relative to the fraction number (Figure 9). This occurred because, as reported in the literature [14], α-terpineol is obtained through the hydration reactions of terpenes. During the production of distilled lime oil, acid-catalyzed reactions of bicyclic hydrocarbons occur, thus increasing terpene alcohols, especially α-terpineol.

**Figure 10:**
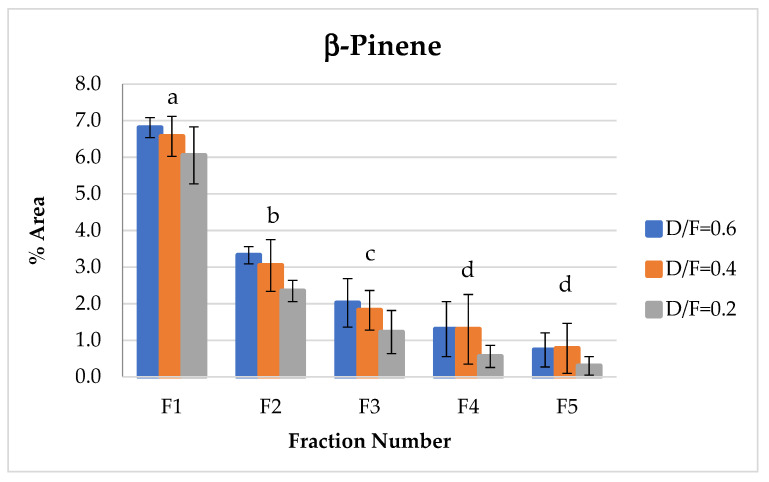
Abundance profile vs. number fraction for β-pinene. The results are the average of three replicates ± standard deviation. Different letters over a column indicate statistical differences (Tukey, *p* < 0.05).

**Figure 11:**
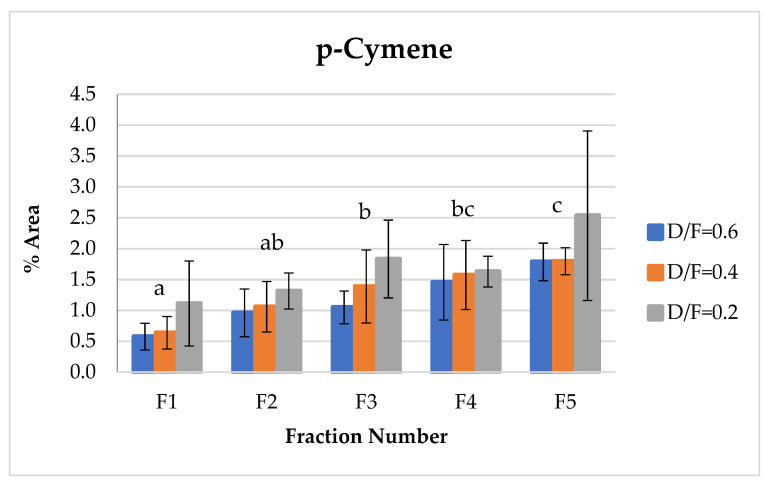
Abundance profile vs. number fraction for p-cymene. The results are the average of three replicates ± standard deviation. Different letters over a column indicate statistical differences (Tukey, *p* < 0.05).

The β-pinene of the group of terpenes showed the same behavior as d-limonene, which decreased in abundance with respect to the fraction number (Figure 10). This decrease in β-pinene was likely due to the transformation of bicyclic monoterpenes to monocyclic monoterpenes during heating.

p-Cymene, which indicates the degree of transformation or degradation of the essential oil product due to contact with steam under acidic conditions at high temperatures, showed an increase relative to the fraction number (Figure 11) because of the dehydrogenation of cyclohexene rings of d-limonene or cyclohexadiene from γ-terpinene [9,14].

p-Cymene is produced via the degradation of γ-terpinene and indicates the oxidation and degradation processes of the essential oil [14]. Therefore, in this study, the p-cymene concentration increased as the dosage of steam increased under acidic pH conditions. For the first fractions, p-cymene presented lower contents in Fractions 2, 3, 4, and 5.

Neral and geranial, in addition to showing significant differences between the different fractions, decreased in abundance relative to the fraction number. The first fraction showed the highest content, which was greater than that from batch distillation, whose content was similar to that of Fraction 3. Neral and geranial decreased because the terpene aldehydes were almost completely lost during distillation [9].

**Figure 12:**
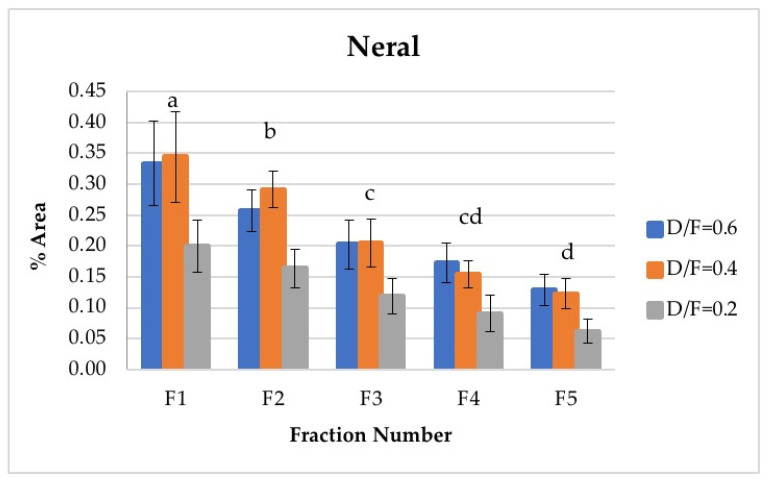
Abundance profile vs. number fraction for neral. The results are the average of three replicates ± standard deviation. Different letters over a column indicate statistical differences (Tukey, *p* < 0.05).

**Figure 13:**
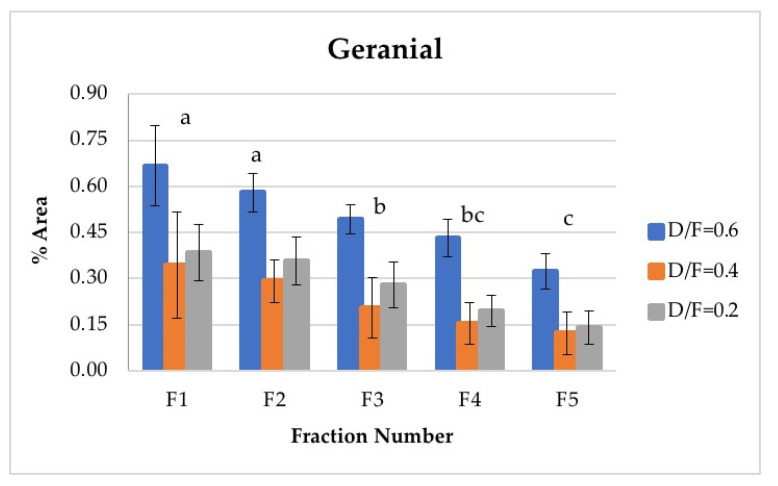
Abundance profile vs. number fraction for geranial. The results are the average of three replicates ± standard deviation. Different letters over a column indicate statistical differences (Tukey, *p* < 0.05).

The citral content (neral + geranial), which is shown in Table 4, agreed with that reported in the literature by other authors [8,16]. The citral content in the first fraction from continuous distillation (Figure 12 and Figure 13) was around 0.95% of the area, and that in the fifth fraction was around 0.30% [8]. It was previously reported that the citral content in the Persian lime *Citrus latifolia* ranges from 0.5% to 2.2%. The value obtained through microwave-assisted hydrodistillation was 0.66% area, distributed among the isomers neral (Z-citral), with 0.29% area, and geranial (E-citral), with 0.38% area [16]. In this way, Fractions 1 and 2 presented a greater quantity of citral than that obtained by microwave-assisted hydro-distillation, whereas Fraction 3 presented a similar quantity of citral to that in the essential oil of Persian lime obtained by batch distillation. No statistically significant differences were found between the experimental runs carried out via batch distillation. (Table 4).

The fractions presented significant differences in the composition of minority compounds able to affect the aroma of essential oil fractions. Further studies are required at other residence times to evaluate the effects of these compounds on composition.

#### 2.4.2. Effect of D/F on the Relative Abundance of Compounds

The D/F had an effect on d-limonene, with statistically significant differences observed at 95% confidence in the abundance of the seven monitored compounds because of the effect of the D/F ratio.

**Figure 14:**
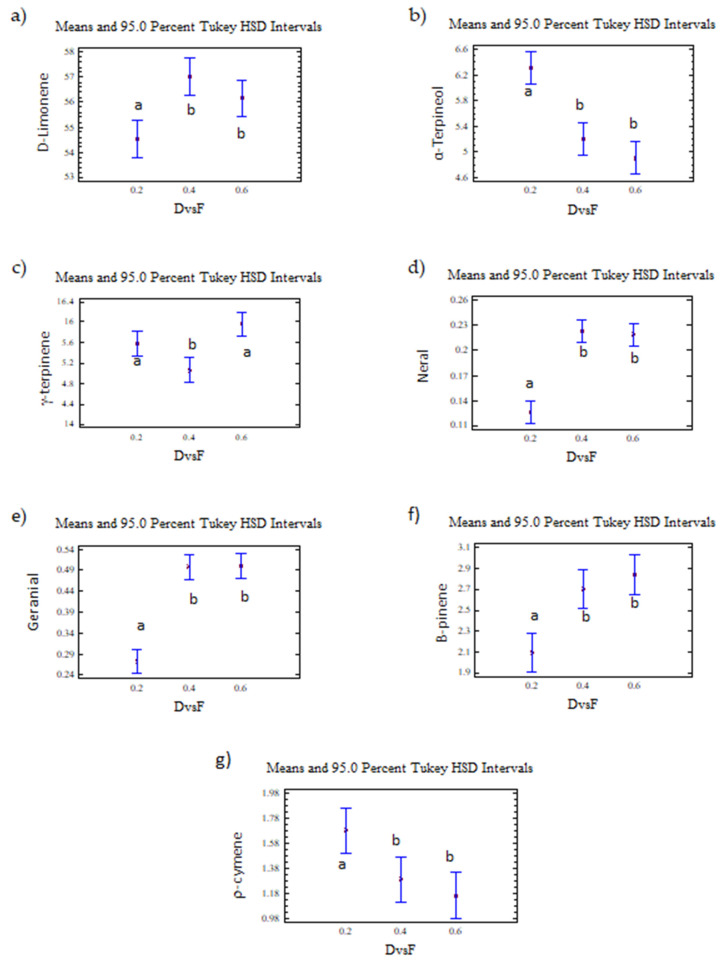
(**a**) d-limonene, (**b**) α-terpineol, (**c**) γ-terpinene, (**d**) neral, (**e**) geranial, (**f**) β-pinene, (**g**) p-cymene. The results are the average of three replicates ± standard deviation. Lowercase letters indicate homogenous groups.

D-limonene, γ-terpinene, α-terpineol, neral, and geranial were presented as two homogeneous groups. The first homogeneous group (group a) included a D/F ratio of 0.2, and the second homogeneous group (group b) included D/F ratios of 0.4 and 0.6 (Figure 14).

On the other hand, the γ-terpinene was presented as two homogeneous groups (Figure 14). The first group (group a) included D/F ratios of 0.2 and 0.6, and the second group (group b) included a D/F ratio of 0.4.

Based on the homogeneous groups obtained in the statistical analysis, the highest relative abundances (% area) of neral and geranial, d-limonene, β-pinene, and α-terpineol were reached with the D/F 0.4 and 0.6 relationships, with no differences observed between these two conditions. Therefore, the process condition that obtained the greatest amount of citral with a reduction in steam consumption was a D/F of 0.4.

With 95% confidence, no statistically significant differences were found in the F1 fractions with respect to the experimental runs carried out in batch distillation (Table 5).

Since some fractions presented concentrations close to those of distilled oil obtained via batch processes, this process was explored under a residence time of four hours, leading to a reduction greater than 50% compared to traditional processes. Furthermore, the juice effluent can be used for the preparation of a rehydrating drink. However, other process conditions must be explored to study the effects of residence time on the composition and oil recovery efficiency. The results suggest that a longer process time may be desirable, as the liquid and vapor contact time increased under acidic conditions and high temperatures. This high-temperature, acidic environment favored the transformation of compounds, thereby impacting the final composition of the distilled oil similar to traditional processes.

In the first stage, it is possible to recover the essential oil contained in the juice. Although it is possible to obtain fractions with different compositions to the studied compounds, more studies are needed to obtain well-differentiated terpene, sesquiterpene, or oxygenated fractions. Greater separation could be achieved by using packed columns in the experimental distillation process. This phenomenon will be explored in future works.

## 3. Methodology

### 3.1. Process

#### 3.1.1. Raw Material

The raw material used for the experimental tests was Persian lime (*Citrus latifolia* Tanaka) from the region of San Martin Hidalgo, Jalisco.

Fresh, green-looking Persian lime juice was used in the initial stages of yellowing and was obtained by pressing the whole fruit using a screw press.

We first ensured that the fruit was well developed, green in color, and had no signs of rot. Each lime was manually washed with water to eliminate any foreign agents. The lime was then processed in a helical press in the pilot plant of CIATEJ. This press has a conical shape and features perforated walls where the juice comes out. The fruit was crushed between these walls to break the cells containing the essential oil, forming a juice–oil emulsion.

#### 3.1.2. Batch Distillation by Steam

The juice–oil emulsion was then transported to the batch distillation equipment described above, where steam was injected to increase the temperature, thus evaporating the water and essential oil. The vapors resulting from the distillation passed through a coil condenser where they changed into a liquid form. This liquid formed by the water and essential oil was deposited into Florentine vessels, where the essential oil was separated by decantation from the water in which it was carried. The recovered essential oil was packaged in amber glass jars, where a small amount of sodium sulfate was added to eliminate any trace of moisture. The amber glass jars containing the essential oil were then stored in refrigeration as a finished product for further analysis.

#### 3.1.3. Continuous Distillation by Steam

Continuous distillation was carried out using the distillation equipment previously described.

Tests were performed in triplicate for each of the experimental stages under the same conditions, which are described later in the experimental design.

#### 3.1.4. Experimental Design for Continuous Distillation

The experimental design was unifactorial 3^1^, where the factor was the ratio of distilled steam flow to feed flow (D/F), with the following levels: 0.2, 0.4, and 0.6.

Response Variables

The response variables evaluated were as follows:

1. The oil recovery efficiency (%);
2. The percentage of steam consumption compared to the traditional process (%).

The steam consumption was determined using a steam flux Vortex Sensor Prowirl 200 (Endress and Hauser) to quantify the amount of steam consumed during distillation (Magnetic Flow Transmitter Meter, Endress and Hauser).

### 3.2. Physical-Chemical Analysis of the Distilled Essential Oil

Once the distillation tests were carried out, both oil fractions obtained from the continuous tests were subjected to the following physicochemical analyses: the determination of the relative density, the determination of the refractive index, and the determination of the rotary power (optical rotation). We obtained these values to determine the oil’s characteristics and compare those characteristics with those of the oil obtained via the traditional method of steam entrainment.

#### 3.2.1. Determination of Relative Density

The relative density was determined based on the French Standard NF T 75-111 in accordance with the international standard [21,22], as published by the International Organization for Standardization. For this analysis, a DMA 35N digital densimeter was used under the conditions specified in the standard.

#### 3.2.2. Determination of the Refractive Index

The refractive index was determined based on the French Standard NF T 75-112 in accordance with the international standard [20], as published by the International Organization for Standardization. A Leica Mark II Plus Abbe Refractometer was used for this analysis.

#### 3.2.3. Determination of the Rotatory Power

To determine the rotatory power, a Poli Science Model SR6 polarimeter was used. This analysis was performed based on the French Standard NF V 75-113 in accordance with the international standard [21], as published by the International Organization for Standardization.

#### 3.2.4. Determination of the Content of Essential Oil in Lime Juice

As in previous analyses, the content of volatile oils in the juice was determined for the raw material according to French Standard NF V 03-409 in technical agreement with the international standard ISO/DIS 6571.

#### 3.2.5. Determination of the Essential Oil Components by Gas Chromatography

The volatile composition of the essential oil or fractions was determined according to the norm AFNOR [21].

Oils in the distilled fractions were analyzed by gas chromatography (GC) using an HP 6890 gas chromatograph equipped with a flame ionization detector (FID). Separation was performed on an HP-1 methyl siloxane capillary column (50 m × 0.2 mm i.d. × 0.33 µm film thickness) with helium as the carrier gas (0.8 mL/min). The oven temperature was programmed initially at 75 °C and was then increased at a rate of 2.5 °C/min to 240 °C. A volume of 0.1 µL was injected at 1:150 split ratio. The injection port and detector temperatures were 260 and 280 °C, respectively. A mixture of air (400 mL/min), hydrogen (40 mL/min), and nitrogen as an auxiliary gas (10 mL/min) was also fed to the detector.

The compounds of interest were identified by the retention time from the injection of pure standards (SIGMA-ALDRICH, purity ≥ 95%) under the same conditions as the sample. The concentration of the compounds was expressed in the % area. Each fraction was analyzed in duplicate.

## 4. Conclusions

The process of continuous steam distillation to obtain essential oil from Persian lime juice presented the highest oil recovery efficiencies (up to 90%) alongside lower steam consumption compared to traditional batch process distillation (up to 50%). More studies are being done to evaluate the application of continuous steam distillation to other essential oils, such as the one from citrus peels. The chemical composition of the oil fractions obtained via continuous distillation showed significant differences for each major component based on the effect of the D/F ratio and the number of the fraction. The chemical compounds that presented the greatest variation in concentration throughout the various fractions were as follows: d-limonene, p-cymene, β-pinene, α-terpineol, and citral (neral + geranial).

## Figures and Tables

**Table 1:** Effect of the D/F and the fraction number in the refractive index, density, and optical rotation.

Main Effects	Refractive Index	Density (g/mL)	Optical Rotation (°)
	*p*-Value	*p*-Value	*p*-Value
D/F	0.0003	0.4452	0.1354
Fraction number	0.0000	0.0004	0.0185

**Table 2:** Reference values for the physicochemical analysis.

Physicochemical Analysis	Continuous Process Fraction 3, D/F = 0.2	Batch Process
Refractive index at 20 °C	1.469 ± 0.0001	1.469 ± 0.00056
Optical rotation (°)	48.875 ± 6.0	45.65 ± 1.6263
Density (g/mL)	0.857 ± 0.0021	0.860 ± 0.00777

**Table 3:** Effect of the D/F ratio on the contents of d-limonene, γ-terpinene, p-cymene, α-terpineol, and β-pinene in the distilled fractions.

Main Effects	d-Limonene	γ-Terpinene	p-Cymene	α-Terpineol	β-Pinene	Neral	Geranial
	*p*-Value	*p*-Value	*p*-Value	*p*-Value	*p*-Value	*p*-Value	*p*-Value
D/F	0.0000	0.7087	0.0000	0.0000	0.0000	0.0000	0.0000
Fraction number	0.0005	0.0001	0.0027	0.0000	0.0000	0.0000	0.0000

**Table 4:** Content of citral isomers (neral and geranial) in Persian lime.

Compound	This Study’s Continuous Processes (% Area)	This Study’s Batch Process (% Area)	Batch Process [8] (% Area)	Microwave-Assisted Hydrodistillation [6] (% Area)
Neral	0.3	0.554 ± 0.240	Neral+ geranial: 0.5 to 2.2	0.29
Geranial	0.6	0.438 ± 0.611		0.38

**Table 5:** Composition of essential oil of Persian lemon by different processes.

Compound	This Study’s Continuous Processes Fraction 1 D/F = 0.4 (% Area)	This Study’s Batch Process (% Area)	Batch Process [8] (% Area)	Microwave-Assisted Hydrodistillation [6] (% Area)
d-limonene	60.09 ± 1.21	55.26 ± 1.75	59.016	61.93
γ-terpinene	14.87 ± 0.58	15.14 ± 0.38	16.089	16.93
α-terpineol	2.76 ± 0.60	2.78 ± 034	2.145	0.39
β-pinene	6.57 ± 0.55	8.17 ± 0.58	6.035	11.35
p-cymene	0.64 ± 0.26	0.64 ± 0.07	1.507	0.21
neral	0.34 ± 0.007	0.55 ± 0.24	0.091	0.29
geranial	0.68 ± 0.17	0.44 ± 0.61	0.121	0.38

## Data Availability

The data presented in this study are available on request from the corresponding author.

## Sample Availability

Samples of the compounds are not available from the authors.
